# Parental Weight Status and Offspring Cardiovascular Disease Risks: a Cross-Sectional Study of Chinese Children

**DOI:** 10.5888/pcd12.140384

**Published:** 2015-01-08

**Authors:** Kayne McCarthy, Yong-ling Ye, Shuai Yuan, Qi-qiang He

**Affiliations:** Author Affiliations: Kayne McCarthy, School of Public Health, Wuhan University, Wuhan, P. R. China and University of Hawaii Office of Public Health Studies, Honolulu, Hawaii; Yong-ling Ye, Shuai Yuan, School of Public Health, Wuhan University, Wuhan, P. R. China.

## Abstract

**Introduction:**

Prevalence of childhood obesity in China is increasing, and parental weight is a risk factor for the development of obesity in children. We examined the relationship of parental body weight status with risk of offspring cardiovascular disease (CVD) in Chinese children.

**Method:**

We conducted a cross-sectional study in Wuhan, China, during May and June 2010. Parental body mass index (BMI) was calculated according to self-reported height and weight. Offspring CVD risk factors, including BMI, waist circumference, blood pressure, fasting glucose, triglycerides, high-density lipoprotein (HDL) cholesterol, low-density lipoprotein (LDL) cholesterol, cardiorespiratory fitness (CRF), and metabolic risk score (MRS), were assessed through anthropometric measures, blood samples, and a CRF test. Multiple linear regression and analysis of covariance were used to examine the effects of maternal and paternal weight status on offspring CVD risks.

**Results:**

A total of 580 Chinese children (339 boys and 241 girls, mean [standard deviation] age, 9.6 [0.7] years) participated in the study. Maternal BMI was significantly associated with offspring elevated BMI (β = 0.134, *P* = .002), waist circumference (β = 0.253, *P* = .04), and decreased CRF (β = −0.134, *P* = .01). Paternal BMI was significantly associated with elevated offspring BMI (β = 0.161, *P* < .001), waist circumference (β = 0.404, *P* < .001), triglycerides (β = 0.017, *P* = .03), MRS (β = 0.084, *P* = .03), and decreased CRF (β = −0.174, *P* < .001). BMI (*P* < .001), waist circumference (*P* < .001), and MRS (*P* < .05) were positively associated with additional overweight/obese parents, whereas CRF was negatively associated (*P* < .001).

**Conclusion:**

Parental weight status was significantly associated with increased risk of CVD in their children, and the association was stronger for paternal weight status.

## Introduction

Overweight and obesity affect 23% of male Chinese youths and 14% of female Chinese youths younger than age 20 (1). Furthermore, the prevalence of childhood obesity in China is increasing, mirroring that of the global community; it has increased nearly tenfold since 1985 (2,3). This immense increase in overweight and obese children and young adults is a threat to public health, because excessive adiposity in childhood is related to a series of physical and mental health problems later in life (4–6).

It is well understood that parental weight is a risk factor for the development of obesity in children (7,8). However, studies have shown conflicting associations between mother’s and father’s weight status on their children’s body mass index (BMI). One study found that the association between maternal BMI and offspring BMI was similar to that of paternal BMI and offspring BMI in a sample of 4,654 complete parent–offspring trios (9). However, another study observed a significantly stronger mother–child association than father–child association for BMI in a group of 7,078 children aged 2 to 15 years (8). Such studies have primarily focused on the effects of parental BMI on their offspring’s BMI (8,9), whereas only a few studies in Europe have looked at the relationship between parental BMI and other cardiovascular disease (CVD) risks in children (10).

Because childhood and adulthood obesity are a result of the interplay between genetic, socioeconomic, and behavioral factors (11), results from Western populations cannot be directly applied to China. Therefore, we conducted a cross-sectional study to examine the association between parental weight status and their offspring’s cardiovascular health. We further compared the influences between maternal and paternal BMI on their children’s CVD risks.

## Methods

### Study participants and methods

This study was conducted in Wuhan, China, during May and June 2010. Details of the study design and methods have been described previously (12,13). Briefly, a representative sample was recruited through a multistage sampling procedure. Districts of Wuhan city were classified as either urban or suburban, and 2 districts were randomly selected in both the urban and suburban districts, for a total of 4. In each selected district, 1 primary school was randomly selected. Students in the third and fourth grades of the selected schools were invited to participate in the study. Written informed consent was obtained from parents of the children. Parents were asked to fill out a questionnaire that included questions about their age, weight, height, and socioeconomic status. Physical measurements of each participating child, including height, weight, waist circumference, blood pressure, and fasting blood samples, were assessed. The Medical Research Ethics Committee of Wuhan University and the University of Hawaii Human Subjects Institutional Review Board approved the study.

### Measures

The children’s height (standing erect without shoes), weight (in light clothes), and waist circumference were measured using standard methods. BMI was calculated by dividing weight (kg) by height squared (m^2^). A trained technician measured systolic blood pressure (SBP) and diastolic blood pressure (DBP) with all children sitting in an upright position for at least 5 minutes. Two measurements were taken in the morning, and the mean was used for data analysis. The mean arterial pressure (MAP) was calculated as follows:

DBP + [(SBP) − DBP)/3]

Pubertal development was assessed by direct observation according to the Tanner stages. Breast development in girls and genital development in boys were used for pubertal classification (14).

CRF was assessed by the 20-minute multistage fitness test (15). This test is a useful measure of cardiorespiratory capacity and is a validated and reliable field test in children and adolescents (16). Subjects were asked to run back and forth on a 20-minute course at a predetermined speed guided by audio signals from a compact disc player. The running speed was set to increase at 0.5 km per hour each minute, from a start speed of 8.5 km per hour. Groups of 6 children were instructed to run at speeds following the audio signal and to complete as many as laps as possible, until they could not cope. The children were stopped when they could no longer follow the signal. Predicted maximum oxygen uptake (VO_2_max) derived from the level (maximal speed) and number of laps in the test was used as a measure of CRF.

Blood samples were taken from the antecubital vein after an overnight fast. Glucose, HDL cholesterol, LDL cholesterol, and triglycerides were analyzed enzymatically at the Wuhan Center for Disease Control and Prevention with a Mairui BS-300 Automatic Analyzer (Mairui High Technologies Corp).

CVD risk factors (waist circumference, MAP, HDL cholesterol, triglycerides, and fasting glucose) were used to compute the Metabolic Risk Score (MRS). First, each risk factor was standardized as follows: standardized value = (value − mean)/standard deviation. The HDL cholesterol scores were multiplied by −1, because HDL cholesterol is inversely related to metabolic risk. Next, the MRS was calculated as the sum of the 5 scores. The scores were continuous measures of metabolic risks; higher scores indicate a poorer profile. This method of constructing the MRS variable has been conducted previously in children and adult populations (17).

### Statistical analysis

Parents’ BMI was calculated according to self-reported height and weight and divided into normal or overweight/obese groups based on Chinese cut-off points (18). Children (n = 580) were categorized into 3 groups as having no overweight/obese parents (n = 317), 1 overweight/obese parent (n = 223), or both overweight/obese parents (n = 40). The characteristic differences among groups were determined by using analysis of variance (ANOVA) and χ^2^ test, where appropriate. Multiple linear regression was used to examine the effects of maternal and paternal BMI on each of the offspring CVD risk factors after adjustment for offspring age, sex, pubertal stage, school district, parental age and education, and parental smoking. Analysis of covariance (ANCOVA) was conducted to compare offspring CVD risk factors among different parental weight statuses (no overweight/obese parents, only overweight/obese mother, only overweight/obese father, and both parents overweight/obese). Statistical analyses were performed using the SPSS statistical package (version 20.0; SPSS Inc).

## Results

Of the 800 children who were invited to participate in the study, 765 (95.6%) agreed to participate. After excluding 185 children with missing data (96 did not return parental questionnaires and 89 did not provide blood samples), 580 were included in the final analysis. Compared with children who were excluded from this study, the participants were slightly younger and had nonsignificantly lower BMI. Offspring age and father’s education differed significantly across groups; stage of puberty, age of father and mother, mother’s education, and parental smoking status did not differ significantly among groups (Table 1). In the unadjusted group comparison, BMI (*P* < .001), waist circumference (*P* < .001), triglycerides (*P* < .001), SBP (*P* = .047), and MRS (*P* < .001) significantly increased with increasing parental weight status while CRF (*P* = .001) decreased (Table 2). 

**Table 1:** Participant Characteristics, Cross-Sectional Study of Child Cardiometabolic Risk in Relation to Parents’ Body Mass Index, Wuhan, China, May and June 2010^a^

Characteristic	No Overweight or Obese Parent (n = 317)	1 Overweight or Obese Parent (n = 223)	2 Overweight or Obese Parents (n = 40)	*P* Value
**Age, y, mean (SD)**	9.5 (0.7)	9.6 (0.7)	9.8 (0.7)	.009
**Sex, n (%)**				
Male	177 (55.8)	137 (61.4)	25 (62.5)	.37
Female	140 (44.2)	86 (38.6)	15 (37.5)	
**Pubertal stage, n (%)**				
Tanner 1	4 (1.3)	4 (1.8)	0	.78
Tanner 2	250 (78.9)	167 (74.9)	33 (82.5)	
Tanner 3	62 (19.6)	52 (23.3)	7 (17.5)	
Tanner 4	1 (0.3)	0	0	
**Father’s age, y, mean (SD)**	38.3 (5.2)	37.4 (3.4)	37.7 (4.2)	.14
**Mother’s age, y, mean (SD)**	35.3 (3.6)	35.4 (3.4)	35.8 (4.2)	.76
**Father’s education, n (%)**				
Primary or below	14 (4.6)	4 (1.9)	2 (5.4)	.02
Middle school	76 (24.9)	69 (32.4)	19 (51.4)	
High school	130 (42.6)	84 (39.4)	11 (29.7)	
College or above	85 (27.9)	56 (26.3)	5 (13.5)	
**Mother’s education, n (%)**				
Primary or below	18 (6.1)	11 (5.3)	2 (5.9)	.14
Middle school	106 (35.9)	71 (34.1)	20 (58.8)	
High school	107 (36.3)	81 (39.0)	10 (29.4)	
College or above	64 (21.7)	45 (21.6)	2 (5.9)	
**Father smokes, n (%)**	194 (61.1)	134 (60.1)	19 (47.5)	.25
**Mother smokes, n (%)**	6 (1.9)	1 (.5)	0	.24

a Some values missing.

**Table 2:** Children’s Cardiovascular Disease Risk Factors, According to Parents’ Body Mass Index, Wuhan, China, May and June 2010

Characteristic	No Overweight or Obese Parent (n = 317)	1 Overweight or Obese Parent (n = 223)	2 Overweight or Obese Parents (n = 40)	*P* Value
	Mean (Standard Deviation)			
Body mass index, kg/m^2^	16.3 (2.5)	17.4 (2.8)	17.5 (3.1)	<.001
Waist circumference, cm	57.9 (7.0)	60.6 (8.2)	61.3 (9.2)	<.001
Glucose, mmol/L	4.4 (0.4)	4.4 (0.4)	4.4 (0.5)	.77
HDL cholesterol, mmol/L	1.2 (0.1)	1.2 (0.1)	1.2 (0.1)	.82
LDL cholesterol, mmol/L	2.4 (0.3)	2.4 (0.3)	2.4 (0.2)	.70
Triglycerides, mmol/L	0.9 (0.4)	1.1 (0.6)	1.1 (0.6)	<.001
Systolic blood pressure, mm Hg	90.4 (10.2)	91.9 (9.3)	94.0 (10.3)	.047
Diastolic blood pressure, mm Hg	58.3 (7.1)	58.2 (7.3)	57.7 (9.4)	.89
Mean arterial pressure	69.0 (7.2)	69.4 (7.2)	69.8 (8.2)	.71
CRF, ml/kg/min	46.3 (3.2)	45.2 (3.5)	45.5 (4)	.001
Metabolic risk score	−0.4 (2.4)	0.4 (2.8)	0.6 (2.8)	<.001

Abbreviation: CRF, cardiorespiratory fitness; HDL, high-density lipoprotein; LDL, low-density lipoprotein.

Offspring BMI was significantly associated with maternal BMI (β = 0.134, *P* = .002) and paternal BMI (β = 0.161, *P* < .001). Similarly, offspring waist circumference was associated with higher maternal BMI (β = 0.253, *P* = .04) and higher paternal BMI (β = 0.404, *P* < .001). Offspring CRF was negatively associated with maternal BMI (β = −0.134, *P* = .01) and paternal BMI (β = −0.174, *P* < .001). Unlike maternal BMI, higher paternal BMI was significantly associated with triglycerides (β = 0.017, *P* = .03) and MRS (β = 0.084, *P* = .03) (Table 3).

**Table 3:** Multiple Linear Regression Examining the Effect of Maternal and Paternal Body Mass Index (BMI) on Offspring Cardiovascular Disease Risk Factors, Wuhan, China, May and June 2010^a^

Characteristic	Maternal BMI			Paternal BMI		
	β	SEM	*P* Value	β	SEM	*P* Value
BMI, kg/m^2^	0.134	0.042	.002	0.161	0.040	<.001
Waist circumference, cm	0.253	0.122	.04	0.404	0.116	<.001
Glucose, mmol/L	0.005	0.006	.35	−0.009	0.005	.10
HDL cholesterol, mmol/L	0.000	0.002	.94	−0.002	0.002	.28
LDL cholesterol, mmol/L	0.003	0.004	.50	0	0.004	.95
Triglycerides, mmol/L	0.004	0.008	.65	0.017	0.008	.03
Systolic blood pressure, mm Hg	0.284	0.157	.07	0.205	0.149	.17
Diastolic blood pressure, mm Hg	0.069	0.116	.55	−0.005	0.110	.96
Mean arterial pressure	0.141	0.114	.22	0.065	0.108	.55
CRF, ml/kg/min	−0.134	0.053	.01	−0.174	0.050	<.001
Metabolic risk score	0.079	0.040	.05	0.084	0.038	.03

Abbreviation: CRF, cardiorespiratory fitness; HDL, high-density lipoprotein; LDL, low-density lipoprotein; SEM, standard error of the mean.

a Adjusted for sex, age, district, puberty stage, parental education, mother or father age, and parental smoking.

For the male offspring and total samples, significant trends (*P* < .05) were present across the 4 parental BMI groups (Figure). For female offspring, BMI and waist circumference measurements demonstrated significant trends (*P* < .05) across the 4 parental BMI groups; however, CRF and MRS trends were not significant. When overweight/obese mother and father only groups were aggregated into 1 group, having 1 overweight/obese parent, female offspring CRF values showed a significantly decreasing trend (*P* < .05). All comparisons of adjusted means with having only an overweight/obese father significantly differed from those who had no overweight/obese parents, except for girls’ MRS (*P* < .05). Girls with only an overweight/obese mother had a significantly higher BMI than girls who had no overweight/obese parents, and boys with 2 overweight/obese parents had a significant higher (*P* < .05) BMI and MRS than boys with no overweight/obese parents. In the total sample, BMI, waist circumference, and MRS were higher for children with 2 overweight/obese parents than for children with no overweight/obese parents.

**Figure:**
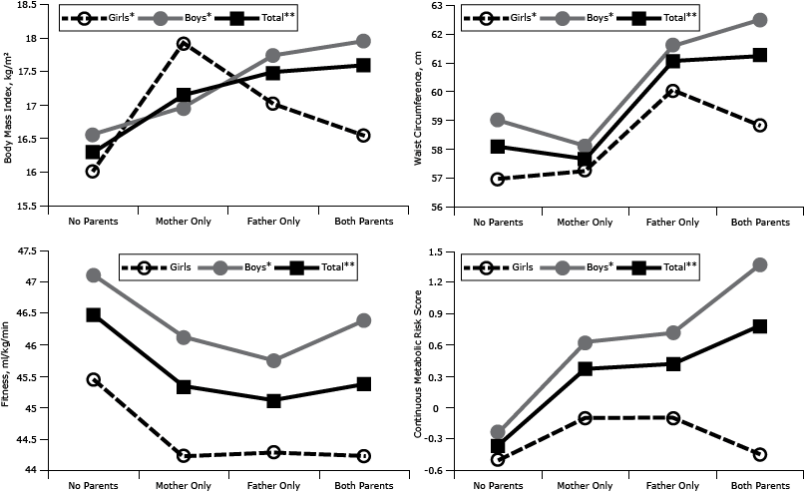
Body mass index (BMI), waist circumference, cardiorespiratory fitness, and metabolic risk score in offspring (girls, boys, total), according to their parents’ BMI classification. Adjustments were made for sex, age, district, parent education, mother’s and father’s age, puberty stage, and parental smoking. Metabolic Risk Score is a continuous measure of metabolic risk; higher scores indicate a poorer profile. *P trend <.05. **P trend < .001. Abbreviations: None, no parents overweight or obese; Mother Only, only mother overweight or obese; Father Only, only father overweight or obese; Both, both parents overweight or obese. **Table:** 

Characteristic	Girls	Boys	Total
**Body mass index**			
None	16.0	16.6	16.3
Mother Only	17.9	17.0	17.1
Father Only	17.0	17.7	17.5
Both Parents	16.5	18.0	17.6
**Waist circumference**			
None	57.0	59.0	58.1
Mother Only	57.3	58.1	57.7
Father Only	60.1	61.6	61.1
Both Parents	58.8	62.5	61.2
**Cardiorespiratory fitness**			
None	45.4	47.1	46.5
Mother Only	44.2	46.1	45.3
Father Only	44.3	45.8	45.1
Both Parents	44.2	46.4	45.4
**Metabolic risk score**			
None	–0.5	–0.2	–0.4
Mother Only	–0.1	0.6	0.4
Father Only	–0.1	0.7	0.4
Both Parents	–0.5	1.4	0.8

## Discussion

We found significant relationships between having overweight/obese parents and offspring CVD risks, including elevated BMI, waist circumference, triglycerides, and MRS, as well as decreased CRF. Maternal BMI was significantly associated with offspring BMI, waist circumference, and CRF; paternal BMI was additionally significantly associated with offspring triglycerides and MRS. These findings suggest that both maternal and paternal weight status are a determinant for childhood adiposity and CVD risk factors. Furthermore, increased paternal weight is a stronger determinant than increased maternal weight for offspring CVD risk factors.

Parental weight status is a risk factor for offspring adiposity development. Furthermore, elevated BMI during the early years of life increases CVD risk factors in later life (19). To the best of our knowledge, only 1 study has examined parent–offspring correlations of blood pressure and BMI in a Chinese population (20). The study found that offspring BMI and blood pressure were significantly associated with parental BMI. Our study extends the evidence by assessing additional CVD risk factors among Chinese children. Similar to our findings, the European Heart Study found that BMI, skinfold thickness, waist circumference, fibrinogen, and SBP increased, while CRF decreased, among children with an increasing number of overweight parents (10).

In this study, the influence of paternal BMI was a stronger determinant than maternal BMI for several offspring CVD risk factors. Although both paternal and maternal BMI were significantly associated with offspring BMI, waist circumference, and CRF, paternal BMI demonstrated influences across all CVD risk factors. In group comparisons, children with overweight/obese fathers demonstrated significantly increased risk factors compared with those who did not have overweight/obese parents. However, girls with overweight/obese fathers had significantly increased BMI and waist circumference but not CRF and MRS.

The higher influence on childhood CVD risks of paternal BMI than maternal BMI may be partly explained by the larger number of overweight/obese fathers (n = 225) than overweight/obese mothers (n = 78) or 2 overweight/obese parents (n = 40) in our study sample. The other explanation is that paternal BMI, although not commonly associated with child body weight during the first 2 years of life, emerges as a determinant for childhood adiposity (21). Previous studies show that paternal BMI is a more predictive indicator for offspring BMI than maternal BMI (22). In an Australian study, paternal obesity was associated with a fourfold increase in risk of obesity at the age of 18 (23).

Because of the late effect of paternal weight on offspring adiposity development, it can be inferred that much of the effects are environmental. Schoolchildren from Sorocaba, Brazil, who lived with their fathers who had a history of kidney disease, heart failure, or hypertension were 76% more likely (odds ratio = 1.76, *P* = .02) than other students to gain excess weight (24). Previous studies have shown that fathers, more than mothers, demonstrate a controlling influence on offspring food consumption (25). This control associated with eating behaviors becomes more permissive with increased paternal BMI (26). Permissiveness toward food control increases offspring risk for gaining weight. In conjunction with an increased control over food consumption, physical activity levels of young children were negatively regulated by increased paternal BMI (22).

Our study has limitations. Our study design was cross-sectional, so findings should be interpreted with caution. Parental height and weight were self-reported and are subject to bias and possible misclassification. However, self-reported height and weight have high sensitivity and specificity among adults younger than 60 (27). Lifestyle factors, such as energy intake and physical activity, were not collected in this study. The study’s small sample size and exclusive location make findings generalizable to children of neighboring Chinese districts and provinces, but caution must be taken when generalizing to national and international child populations. Regardless of these limitations, this study is among the few to provide insight into intergenerational influences on several offspring CVD risk factors in China, while controlling for important potential confounders.

Our results indicate that parent weight status was significantly associated with increased risk of CVD in their offspring, and the association was stronger for paternal weight status. The findings highlight the importance of preventive interventions that target parental obesity, especially paternal BMI, as a holistic familial approach to reduce children’s CVD risk.
